# GPCRomics: GPCR Expression in Cancer Cells and Tumors Identifies New, Potential Biomarkers and Therapeutic Targets

**DOI:** 10.3389/fphar.2018.00431

**Published:** 2018-05-22

**Authors:** Paul A. Insel, Krishna Sriram, Shu Z. Wiley, Andrea Wilderman, Trishna Katakia, Thalia McCann, Hiroshi Yokouchi, Lingzhi Zhang, Ross Corriden, Dongling Liu, Michael E. Feigin, Randall P. French, Andrew M. Lowy, Fiona Murray

**Affiliations:** ^1^Department of Pharmacology, University of California, San Diego, San Diego, CA, United States; ^2^Department of Medicine, University of California, San Diego, San Diego, CA, United States; ^3^Department of Pharmacology and Therapeutics, Roswell Park Comprehensive Cancer Center, Buffalo, NY, United States; ^4^Department of Surgery, University of California, San Diego, San Diego, CA, United States; ^5^Moores Cancer Center, University of California, San Diego, San Diego, CA, United States; ^6^School of Medicine, Medical Sciences and Nutrition, University of Aberdeen, Aberdeen, United Kingdom

**Keywords:** breast cancer, cancer microenvironment, chronic lymphocytic leukemia, colon cancer, GPCR array, orphan receptors, pancreatic cancer

## Abstract

G protein-coupled receptors (GPCRs), the largest family of targets for approved drugs, are rarely targeted for cancer treatment, except for certain endocrine and hormone-responsive tumors. Limited knowledge regarding GPCR expression in cancer cells likely has contributed to this lack of use of GPCR-targeted drugs as cancer therapeutics. We thus undertook GPCRomic studies to define the expression of endoGPCRs (which respond to endogenous molecules such as hormones, neurotransmitters and metabolites) in multiple types of cancer cells. Using TaqMan qPCR arrays to quantify the mRNA expression of ∼340 such GPCRs, we found that human chronic lymphocytic leukemia (CLL) cells/stromal cells associated with CLL, breast cancer cell lines, colon cancer cell lines, pancreatic ductal adenocarcinoma (PDAC) cells, cancer associated fibroblasts (CAFs), and PDAC tumors express 50 to >100 GPCRs, including many orphan GPCRs (which lack known physiologic agonists). Limited prior data exist regarding the expression or function of most of the highly expressed GPCRs in these cancer cells and tumors. Independent results from public cancer gene expression databases confirm the expression of such GPCRs. We propose that highly expressed GPCRs in cancer cells (for example, GPRC5A in PDAC and colon cancer cells and GPR68 in PDAC CAFs) may contribute to the malignant phenotype, serve as biomarkers and/or may be novel therapeutic targets for the treatment of cancer.

## Introduction

Knowledge regarding the biology of tumors and malignant cells has greatly expanded in recent years. Several hallmarks of cancer have been identified: proliferative signaling, replicative immortality, evasion of growth suppressors, resistance to cell death, induction of angiogenesis, and the activation of invasion and metastasis (Hanahan and Weinberg, 2011). A key recent focus for basic and clinical investigators has been the identification of “driver mutations,” including ones that are shared among anatomically distinct types of cancer and predicted to be responsive to molecularly targeted therapeutics. In parallel has been the growth of personalized (precision) medicine approaches guided by genetic analyses that seek to identify such driver mutations (Schwaederle et al., 2015; Borad and LoRusso, 2017; Chae et al., 2017; Hyman et al., 2017). In addition, increased understanding of the immune suppression that contributes to tumor growth and metastasis and development of therapeutics directed at this immune suppression have yielded improved clinical outcomes for a variety of cancers (Cogdill et al., 2017; Gotwals et al., 2017; Lim and June, 2017).

In spite of such progress, new therapies are needed for most cancers. In this regard, GPCRs, the largest family of signaling receptors in humans and the largest family of protein targets for approved drugs (Sriram and Insel, 2018), have rarely been exploited as therapeutic targets in cancer with the exception of certain endocrine cancers (e.g., pituitary, adrenal, testes, ovarian) and hormone-responsive tumors (e.g., breast and prostate cancer). Even though GPCRs are not thought to be functionally mutated and commonly expressed (i.e., they are not “genetic drivers”) in cancers, GPCRs and post-GPCR signaling mechanisms play an important role in regulating cellular functions integral to the hallmarks of cancer (e.g., growth/proliferation, metabolism, death/apoptosis, ion and nutrient transport, and migration; Dorsam and Gutkind, 2007; Hanahan and Weinberg, 2011; O’Hayre et al., 2014). GPCRs are not only expressed by cancer cells themselves, but also by cell-types in the tumor microenvironment, including stromal (fibroblast), vascular, immune, and inflammatory cells. Numerous effects of GPCRs, including their regulation of apoptotic cell death, are mediated by the second messenger cAMP (Insel et al., 2012, 2014). Moreover, as plasma membrane proteins, GPCRs should be targetable as are numerous other types of cell surface proteins in various cancers.

The lack of consideration of GPCRs as therapeutic targets may relate, at least in part, to the limited information regarding their expression by cancer cells and cells of the tumor microenvironment. We therefore have used an unbiased (GPCRomic) approach to identify and quantify GPCR expression in cancer cells in order to identify GPCRs that may contribute to the malignant phenotype and might be therapeutic targets. Here, we present findings that utilize GPCR-specific PCR-based arrays, RNA-seq, and mining of databases to define GPCR expression of primary cancer cells, cancer cell lines, cells in tumor tissue, and the tumor microenvironment. The findings reveal previously unrecognized GPCR expression in these cells suggesting that they could serve as novel biomarkers and therapeutic targets in various types of cancer.

## Materials and Methods

### Primary Cells and Cell Lines

**Table 1** summarizes the cells used in the current studies and the source from which they were obtained. Patient cells were isolated after informed consent, under an Institutional Review Board-approved protocol, and within the guidelines of the Health Insurance Portability and Accountability Act. All cells were grown under standard tissue culture conditions and studied when growing at log phase and at low passage number.

**Table 1:** Human cells and cell lines used for assessment of GPCR expression.

Type of cancer	Cells or cell lines used	Source of cells or cell lines
B-cell chronic lymphocytic leukemia (CLL)	Patient-derived primary cells Normal human B cells	UCSD Moores Cancer Ctr
Bone marrow stromal natural killer (NK) cells that support CLL cell growth	NK cells immortalized with telomerase reverse transcriptase	UCSD Moores Cancer Ctr
Colon cancer	T-84, Caco-2 cell lines	ATCC
Triple-negative breast cancer	BT-20, HS-578, MDA-MB-157, MDA-MB-436 cell lines	ATCC
Pancreatic ductal adenocarcinoma	34E/79E patient-derived cells (PDAC) cells and PDAC tumors Human control pancreatic ductal epithelial cells AsPC-1, MiaPaCa-2 cell lines	UCSD Moores Cancer Ctr. ATCC
Pancreatic ductal adenocarcinoma	5 primary patient-derived CAFs Human PSCs: Corresponding “normal” precursor cells	Lowy lab, UCSD Moores Cancer Ctr ScienCell Research Laboratories (#3830; ScienCell Research Laboratories, Carlsbad, CA, United States)


### Isolation of mRNA and Preparation of cDNA

RNA was isolated using a Qiagen RNeasy MiniKit, with on-column DNase-1 digestion (Qiagen, Valencia, CA, United States). cDNA was synthesized using a Superscript III First Strand Synthesis Kit (Invitrogen, Carlsbad, CA, United States) as per the manufacturer’s instructions, using random hexamer priming.

### Analysis of GPCR Expression

**FIGURE 1:**
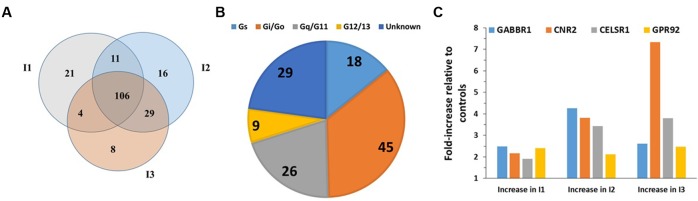
GPCR expression in B-CLL cells. **(A)** Venn diagram showing the number of GPCRs expressed in B-CLL cells (I1, I2, and I3) isolated from three different patients with indolent CLL. I1, I2, and I3, expressed 142, 162, and 147 GPCRs, respectively, and shared in expression of 106 GPCRs. **(B)** G protein linkages of the 106 commonly expressed GPCRs. **(C)** The expression (ΔCt normalized to 18S rRNA) and fold-increase in expression in indolent CLL cells compared to control B cells of the four GPCRs (GABBR1, CNR2, CELSR1, and GPR92) with approximately twofold (or greater) increases in expression in the CLL cells. *n* = 3 biological replicates of B-CLL, analyzed on one array each.

We used TaqMan GPCR arrays (Thermo Fisher Scientific) to identify and quantify GPCR expression (Snead and Insel, 2012). The Taqman GPCR arrays for humans contain primers for the majority (340) of GPCRs in peripheral tissues that have endogenously expressed ligands (i.e., endoGPCRs), including all well characterized GPCRs which are targets for approved drugs (Sriram and Insel, 2018). mRNAs for only seven olfactory receptors, one taste receptor, and three vision receptors are detectable by these arrays. For each array, ∼1 μg of total RNA converted to cDNA was loaded into a 384-well microfluidic card along with TaqMan Universal PCR Master Mix according to the manufacturer’s instructions. Exponential curves for each detected cDNA were analyzed using RQ manager software. GPCR expression was normalized to the Ct of 18S rRNA (whose Ct was generally ∼12). Data are expressed as ΔCt = Ct_x_ - Ct_18S_ for each GPCR (x) detected. We typically used a ΔCt of 25 as the GPCR detection threshold, a value similar to that in prior studies (Snead and Insel, 2012; Amisten et al., 2015; Walker et al., 2015; Jung et al., 2016). Independent qPCR analyses consistently confirmed the expression of array-identified GPCRs. We also found excellent concordance between GPCR data from TaqMan arrays and RNA-seq. We assigned G protein linkage of GPCRs based on information for the primary or multiple linkages in the IUPHAR/British Pharmacological Society (BPS) GtoPdb (Alexander et al., 2017). Certain GPCRs reportedly have multiple such linkages but this may depend on the cells studied and on the use of transfection/over-expression approaches, which may not accurately reflect signaling in cells *in vivo*. The patterns of G protein linkages we report should therefore be considered tentative. In figures that show the number of GPCR linkages (e.g., **Figure 1**), we include all reported linkages for a given GPCR. Hence, the number of linkages indicated is generally greater than the number of GPCRs identified. RNA-seq data on pancreatic CAFs were generated as described in Wiley et al. (2018). In brief, Truseq stranded mRNA libraries were sequenced at ∼25 million 75 bp single reads per sample, on an Illumina NextSeq 500 sequencer by DNA Link, Inc. (San Diego, CA, United States). Data were analyzed via alignment using STAR (Dobin et al., 2013) and quantification via Cufflinks (Trapnell et al., 2012) with the hg38 reference genome and refseq annotations; and subsequently via edgeR (Robinson et al., 2010) to facilitate comparison of gene expression between samples. Accession number for RNA-seq data: GSE101665.

### Data Mining and Analysis

RNA-seq data for normal pancreas from the GTEx database (GTEx Consortium, 2013) and pancreatic tumors from TCGA (Weinstein et al., 2013) were downloaded from the Xena portal^1^ from data generated by the TOIL pipeline (Vivian et al., 2017). Data were generated using alignment via STAR (Dobin et al., 2013), and quantification via RSEM (Li and Dewey, 2011), using the hg38 reference genome and Gencode V23 annotations^2^. Gene-level RSEM estimated counts for normal pancreas (*n* = 165) and pancreatic adenocarcinoma (PAAD, *n* = 179 tumors plus four matched normal in TCGA) were downloaded, along with information regarding phenotype. The histology of 147 of the 179 tumors was consistent with PDAC; thus we compared the expression data in those 147 tumors with that of normal pancreas. The counts matrix with GTEx and TCGA samples was analyzed via edgeR (Robinson et al., 2010) using TMM normalization to obtain expression in counts per million (CPM). Exact testing was used to evaluate differential expression. We used the batch correction tool in Limma (Smyth, 2005) to verify that factors such as plate identity, sequencing center or source collection center (as relevant variables^3^) had minimal impact on GPCR expression. GPCR expression was extracted by querying expression of genes corresponding with annotated GPCR gene names from the GtoPdb database (Alexander et al., 2017).

We determined GPCR expression in cancer cell lines from the EBI database (Kapushesky et al., 2009) containing analyzed samples via the iRAP pipeline^4^ (Fonseca et al., 2014), yielding gene expression in FPKM, as computed by Cufflinks on aligned BAM files generated using Tophat2 (Trapnell et al., 2012) with GRCh37.66 from Ensembl as the reference human genome.

We set the detection threshold for GPCRs as >0.1 FPKM, as used previously (Chettoor et al., 2014; Zhang et al., 2014), which yields results comparable to the ΔCt = 25 threshold of the TaqMan array data. GPRC5A expression in PDAC cell lines assayed via RNA-seq was normalized to β-actin (ACTB) for comparison with TaqMan array data and to facilitate comparison of our GPRC5A expression data in control PDECs with the EBI data for PDAC cell lines. Use of other housekeeping genes (e.g., GAPDH, β2 microglobulin) did not alter our conclusions.

### Immunocytochemistry for Detection of GPRC5A

BXPC-3 and MIA PaCa-2 cells (pancreatic cancer cell lines that express GPRC5A mRNA) were plated on cover slips at ∼50% confluency and fixed using 4% paraformaldehyde, 24 h after plating. Cells were stained with GPRC5A primary antibody HPA007928 from Sigma Aldrich, United States, based on protocols provided by the manufacturer, followed by 1 h incubation with secondary goat-anti rabbit antibody (cat # A-11008, Invitrogen, United States). Cells were also stained with DAPI (4′,6-diamidino-2-phenylindole) to visualize nuclei. Images were then taken via a Keyence BZ-X700 microscope and analyzed using ImageJ (Schneider et al., 2012).

## Results

Limited information exists regarding the profile of GPCRs expressed by malignant cells. Prior studies primarily assessed individual GPCRs, in terms of expression, signaling and functional activities (Lappano and Maggiolini, 2011; Feigin, 2013; O’Hayre et al., 2014; Bar-Shavit et al., 2016; Liu et al., 2016; Van Jaarsveld et al., 2016). TaqMan GPCR arrays provide an unbiased method to identify and quantify non-chemosensory GPCRs (other than those for taste, olfaction, and vision). These arrays include ∼340 endoGPCRs (GPCRs that respond to endogenously expressed hormones, neurotransmitters, autocoids and metabolites), ∼120 of which are orphan GPCRs (i.e., without known physiologic agonists). Data for individual mRNAs as a PCR Ct is compared to the Ct for a housekeeping gene (e.g., 18S rRNA) to yield a ΔCt value. Since Ct values are inversely related to expression, lower Ct values indicate higher mRNA expression.

### Human B-CLL Cells and Control B Cells

B-cell Chronic Lymphocytic Leukemia, the most common adult leukemia in economically developed countries, is characterized by the accumulation of mature B cells, primarily as a consequence of reduced apoptosis (Billard, 2014; Hallek, 2015). CLL is clinically quite variable: patients with CLL can survive a few months (aggressive CLL) or many years (indolent CLL) after diagnosis (Nabhan and Rosen, 2014). Increases in cAMP can promote apoptosis in CLL cells (Murray and Insel, 2013). Analysis of malignant B cells from three patients with indolent CLL identified 106 commonly detected (including 30 orphan) GPCRs (**Figure 1A**). Most highly expressed GPCRs (ΔCt < 18) were expressed by all three patients. More of these GPCRs couple to Gi than to other G proteins but 31 GPCRs have unknown G protein linkages (**Figure 1B**). The highest expressed Gi-coupled receptors include CXCR4, EBI2, and CCR7 (ΔCt values of 12.2, 12.7, and 14.5, respectively). The highest expressed Gs-coupled receptors include ADRB2 (β2-adrenergic receptor, ΔCt = 14.6) and PTGER4 (ΔCt = 14.9). Control B cells express 175 GPCRs, including 101 of the 106 CLL-expressed GPCRs. B-CLL cells have a greater than twofold increase in expression of a subset of GPCRs that are highly expressed (ΔCt ∼18 or less) in normal B cells (**Figure 1C**): GABBR1 (GABA B1 receptor, ΔCt = 16), CNR2 (CB_2_ cannabinoid receptor, ΔCt = 17), CELSR1 (an orphan adhesion receptor, ΔCt = 17), and GPR92 (aka LPAR5, a lysophosphatidic acid receptor, ΔCt = 18).

### Human Bone Marrow Stromal Natural Killer Cells (BMNK Cells)

**FIGURE 2:**
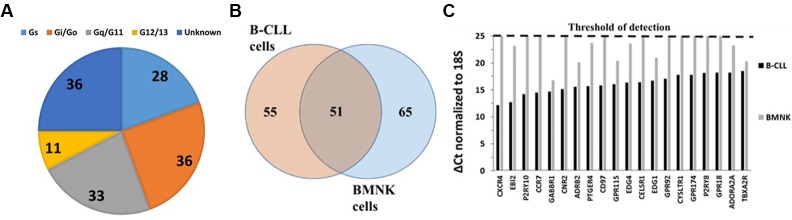
GPCR expression in BMNK cells. **(A)** G protein linkages of the 116 GPCRs detected in BMNK cells. **(B)** Venn diagram of GPCRs expressed in B-CLL compared to BMNK cells, indicating that 51 GPCRs are shared but most receptors are unique for each cell-type. **(C)** Lower expression in the BMNK cells (higher ΔCt values) for most of the highest expressed GPCRs in B-CLL cells (lower ΔCt values). *n* = 1 for each cell type.

Bone marrow stromal natural killer cells, immortalized by expression of human telomerase reverse transcriptase, enhance the viability of primary cultures of B-CLL cells and are considered an important component of the CLL microenvironment (Zhang et al., 2012; ten Hacken and Burger, 2014). BMNK cells express 116 GPCRs, including 33 orphan GPCRs. The 20 highest expressed GPCRs include seven orphan GPCRs [e.g., GPR176 (ΔCt = 14.9), LPHN2 (ΔCt = 15.5), CD97 (ΔCt = 16.1)]. Gi-coupled GPCRs are the largest category of GPCRs for which G protein linkage is known (**Figure 2A**). Such GPCRs include a number of the highest expressed receptors, including F2R (protease activated receptor 1, PAR1, ΔCt = 15.7), LPAR1 (also known as EDG2; ΔCt = 16.3), and SSTR1 (somatostatin receptor-1, ΔCt = 17.7). PAR1 and LPARs both couple to Gq and G12/13.

To determine the similarity in GPCR expression of CLL cells and cells of their tumor microenvironment, we compared the GPCR profiles of B-CLL and BMNK cells. Although 51 of the 106 shared GPCRs among B-CLL cells are also detected in BMNK cells (**Figure 2B**), most of the highest expressed GPCRs in B-CLL cells are either undetectable or expressed at much lower levels in BMNK cells: 103 of the 106 commonly detected receptors in B-CLL cells were more than twofold higher expressed than in BMNK cells and almost all of the highly expressed GPCRs in B-CLL cells have much lower expression in BMNK cells (**Figure 2C**).

### Human Breast Cancer Cells

**FIGURE 3:**
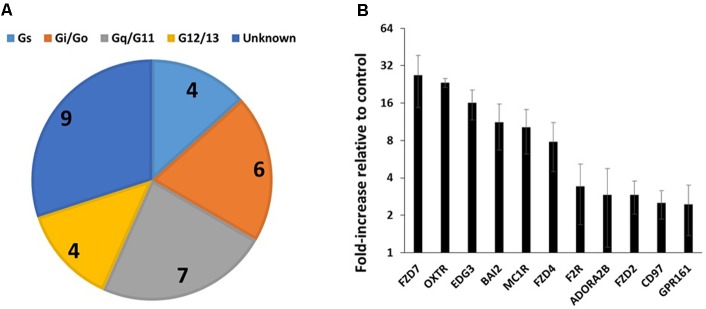
GPCR expression in triple-negative breast cancer cells. **(A)** G protein linkages of the 23 commonly detected GPCRs in four breast cancer cell lines. **(B)** Average (and SEM) of the fold-increases in expression of GPCRs in triple-negative breast cancer cells compared to control cells. Of the 23 commonly detected GPCRs, the 11 shown have a greater than twofold average increase in expression. *n* = 4 cell lines, assayed on one array each.

We assessed GPCR expression in four human triple-negative breast cancer cell lines (BT-20, HS-578, MDA-MB-157, and MDA-MB-436) and a control cell line (MCF-10A, a breast epithelial cell line). The four breast cancer cell lines expressed on average 88 GPCRs and had shared expression of 23 GPCRs (**Table 2**), 21 of which were also detected in the control cell line. Gq-coupled GPCRs are more frequent among the GPCRs with known G protein linkages (**Figure 3A**). **Figure 3B** shows the fold-increases in expression for 11 of the commonly expressed GPCRs with a greater than twofold increase in expression compared to the control breast epithelial cell line. We detected two receptors uniquely expressed in all breast cancer cell lines but not in control cells: GPRC5B (a Class-C orphan receptor, ΔCt = 17.8) and TBXA2R (thromboxane A2 receptor, ΔCt = 19.4).

**Table 2:** GPCR expression in Triple-negative breast cancer cells: The 23 commonly detected GPCRs in four triple-negative breast cancer cell lines with expression (ΔCt) normalized to 18S rRNA.

Gene name	ΔCt vs 18S	Gene name	ΔCt vs 18S	Gene name	ΔCt vs 18S
FZD6	16.1	GPR126	17.7	P2RY11	18.4
CD97	16.6	GPRC5B	17.8	FZD1	18.4
GPR153	16.8	OXTR	17.9	BAI2	18.6
FZD4	16.9	LPHN2	18.0	GPR161	18.7
FZD2	17.2	FZD7	18.0	TBXA2R	19.4
F2R	17.4	GABBR1	18.0	MC1R	19.5
ADORA2B	17.5	GPR125	18.1	GPR135	20.7
OPN3	17.6	EDG3	18.3		


**Table 2** shows the identities and average expression values (ΔCt normalized to 18S rRNA) of the 23 commonly expressed GPCRs in the four breast cancer cell lines. Five of these receptors (BAI2, GPR125, CD97, GPR126, and LPHN2) are members of the adhesion family of GPCRs. Of note, GPR161, an orphan receptor with more than twofold increase in expression, has previously been reported to contribute to the malignant phenotype in triple-negative breast cancer (Feigin et al., 2014).

Analysis of 47 invasive breast carcinomas, including triple-negative cancer cell lines, in the Cancer Cell Line Encyclopedia (CCLE, Barretina et al., 2012, hosted at the EBI expression atlas, Kapushesky et al., 2009) revealed that the cell lines express on average ∼107 GPCRs and highly express several GPCRs identified in our studies above, including CD97, OXTR, ADORA2B, and FZD7. As in the GPCR array studies, the cell lines shared in the expression of relatively few GPCRs: only 19 (including CD97, MC1R, BAI2, FZD1, P2RY11, OPN3, GPR153, and FZD4 in **Table 2**) were detected in all 47 cell lines. The CCLE cell lines include 16 triple-negative cancers (including four cell lines we assessed using GPCR arrays). Of the 23 commonly detected GPCRs in **Table 2**, 19 were detectable in all 16 CCLE triple-negative breast cancer cell lines; four other GPCRs (LPHN2, GPRC5B, EDG3, TBXA2R) were detected in 11–14 of the 16 cell lines. Hence, the GPCRs highlighted in **Table 2** may be ones that contribute to triple-negative breast cancer. Of note, GPR161 is detected in all 16 triple-negative cell lines but only 24 of 31 cell lines from non-triple-negative cancers. With a higher detection threshold (1 FPKM), GPR161 is detected in 12 of 16 triple-negative cell lines but only 13 of 31 other breast cancer cell lines. On average, GPR161 is ∼1.6-fold higher expressed among the triple negative cancer cell lines than in non-triple-negative cell lines.

### Human Colon Cancer Cell Lines

**FIGURE 4:**
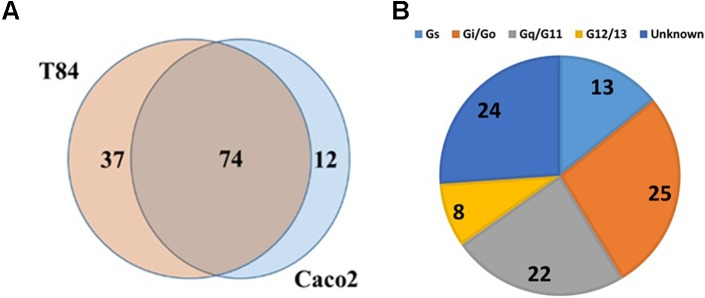
GPCR expression in colon cancer cells. **(A)** Venn diagram showing GPCRs expressed in two colon cancer cell lines, Caco-2 and T84 with 74 GPCRs commonly detected between the two cell lines. **(B)** G protein linkage of the 74 commonly detected GPCRs. *n* = 2 cell lines, assayed on one array each.

TaqMan array analysis of T-84 and Caco-2 colon adenocarcinoma cell lines revealed that they share in expression of 74 GPCRs (**Figure 4A**), including 24 Gi-, 23 Gq- and 11 Gs-coupled receptors (**Figure 4B**). F2RL1 (PAR2, ΔCt = 15.3) is the highest expressed Gq-coupled GPCR. Among the highest expressed Gs-coupled GPCRs are adenosine A2B (ADORA2B, ΔCt = 17.6) and vasoactive intestinal peptide receptor 1 (VIPR1, ΔCt = 17.8). The highest expressed Gi-coupled receptors are OXER1 (oxoeicosanoid receptor 1/GPR170, ΔCt = 17.3) and EDG4/LPAR2 (lysophosphatidic acid receptor 2, ΔCt = 17.3). Of the 74 commonly detected receptors, 18 are orphans; GPR160 is the highest expressed orphan GPCR.

Data mining of GPCR expression in 41 colon adenocarcinoma cell lines in the CCLE (Kapushesky et al., 2009; Barretina et al., 2012) yielded results consistent with those findings. On average, the cell lines expressed 101 GPCRs of which 21 were commonly expressed. F2R/PAR1 was the highest expressed Gi-coupled GPCR (detected in all 41 cell lines). ADORA2B, the highest expressed Gs-coupled GPCR, was detected in 39 cell lines. F2RL1 was the highest expressed Gq-coupled GPCR and overall, the second highest expressed GPCR. Of note and relevant to data for pancreatic cancer (see below), GPRC5A, an orphan receptor, was the highest expressed GPCR, detected in 40 of 41 colon adenocarcinoma cell lines and the highest expressed GPCR in 27 of the 41 cell lines.

### Human Pancreatic Cancer Cells

**FIGURE 5:**
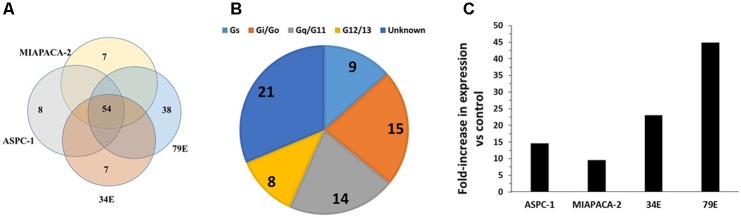
GPCR expression in pancreatic ductal adenocarcinoma (PDAC) cells. **(A)** Venn diagram showing GPCRs expressed in two PDAC cell lines (MiaPaCa, AsPC-1) and primary PDAC cells (34E, 79E); 54 GPCRs were detected in all four samples. The number of GPCRs in each cell-line was: MiaPaCa-2: 92, AsPC-1: 114, 79E: 175, 34E: 130. **(B)** G protein linkage of the 54 commonly detected GPCRs. **(C)** Fold-increase in GPRC5A expression in PDAC cells compared to the control pancreatic ductal epithelial cells (PDEC). *n* = 4 cell lines, each assayed on one array each.

Using TaqMan GPCR arrays, we assessed GPCR expression of pancreatic cancer (PDAC) cell lines (AsPC-1 and MiaPaCa-2), patient-derived primary PDAC cells (identified as 34E and 79E in **Figure 5A**), and a normal pancreatic ductal epithelial (PDEC) cell line. We found that PDAC and PDEC cells express ∼100 GPCRs, of which 54 (including 20 orphan GPCRs) were expressed in all four PDAC samples (**Figure 5A**). Those shared GPCRs included most of the highly expressed GPCRs in each cell line. Gi-coupled and Gq-coupled receptors are the largest groups of GPCRs with known G protein linkages (**Figure 5B**).

GPRC5A was the highest expressed GPCR of the 54 receptors commonly expressed by the PDAC cells and had >10-fold higher expression than any other GPCR. Compared to control PDEC cells, GPRC5A had the greatest fold-increase (>eightfold) in expression in all four PDAC cell samples with larger fold-increases in the primary cancer cells, 34E and 79E (**Figure 5C**).

### Human Pancreatic Cancer Tumor Tissue

**FIGURE 6:**
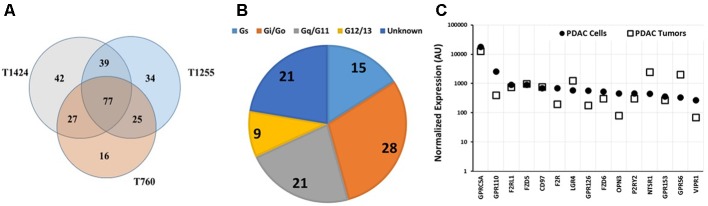
GPCR expression in pancreatic cancer tumors. **(A)** Venn diagram of GPCR expression, determined using Taqman GPCR arrays, of three PDAC tumor samples with 77 GPCRs commonly detected among the samples. T1424, T1255, and T760 expressed 185, 175, and 145 GPCRs, respectively. **(B)** G-protein linkage of the 77 commonly detected GPCRs. **(C)** GPRC5A is expressed at similar levels in PDAC cells and tumors. The highest expressed GPCRs in cancer cells were also highly expressed in tumors. *n* = 3 primary tumors, each assayed on one array each.

TaqMan GPCR array analysis of three PDAC tumors revealed that they share in expression of 77 GPCRs (**Figure 6A**), 16 of which are orphan receptors. The tumors expressed ∼150 or more GPCRs (i.e., more than did the PDAC cell lines, **Figures 5**, **6**), perhaps because of the heterogeneous cell populations and their diverse GPCR repertoires in the tumors. The G protein coupling of tumor-expressed GPCRs was similar to that of PDAC cells (Cf. **Figures 5B**, **6B**): Gi- and Gq-coupled GPCRs were the largest categories of receptors with known G protein coupling. Most of the highest expressed GPCRs were commonly expressed among the tumor samples.

As in the PDAC cells, GPRC5A was the highest expressed GPCR in the tumor samples (∼fourfold more highly expressed than any other GPCR). GPRC5A, an orphan GPCR, may couple to Gi_._ (Hirano et al., 2006). Previous work suggested a role for GPRC5A in various cancers (Zhou and Rigoutsos, 2014). Zhou et al. (2016) have also noted high expression of GPRC5A in PDAC and found that GPRC5A may contribute to cell proliferation, survival, and drug-resistance in PDAC tumors.

### Human Pancreatic Cancer-Associated Fibroblasts (CAFs)

Cancer-associated fibroblasts, an abundant cell type in pancreatic tumor microenvironment, contribute to the extensive fibrotic stroma (desmoplasia) in PDAC. We sought to assess GPCR expression in CAFs to investigate if (a) GPCRs may regulate their pro-fibrotic phenotype and (b) GPCRs contribute to the interaction between CAFs and PDAC cells.

We isolated CAFs from five PDAC patient tumors and assessed GPCR expression in the CAFs by both RNA-seq and Taqman GPCR arrays (Wiley et al., 2018), which allowed us to compare results with the two methods. We also assessed GPCR expression in normal PSCs, precursor cells for CAFs, to determine if GPCR expression changes after PSCs convert to CAFs.

**FIGURE 7:**
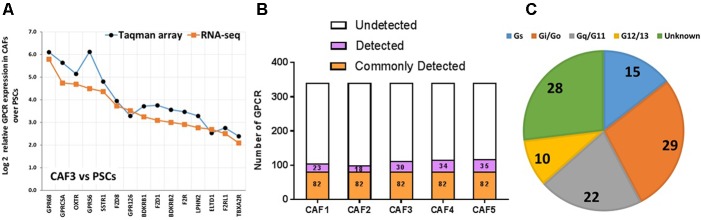
GPCR expression in CAFs. **(A)** Increase in expression, as determined by Taqman GPCR arrays or RNA-seq in one CAF sample (CAF3) compared to pancreatic stellate cells (PSCs) of the 15 GPCRs with the largest increases in expression in CAFs compared to PSCs. **(B)** CAFs express ∼110 GPCRs, with 82 receptors commonly detected in five patient replicates tested. **(C)** G protein linkage of the 82 commonly detected GPCRs. Five separate CAF biological replicates were analyzed via Taqman arrays, while three of these were also analyzed via RNA-seq.

Taqman GPCR arrays and RNA-seq detected ∼110 GPCRs in CAFs and yielded similar results for identities of detected GPCRs and fold-changes in expression between CAFs and PSCs (**Figure 7A** and Wiley et al., 2018). The 82 GPCRs commonly detected by Taqman arrays of five CAF samples (**Figure 7B**) link to various Gα proteins (**Figure 7C**), most frequently to Gq/11 and Gi/Go, and include 28 orphan GPCRs. F2R/PAR1 was the highest expressed GPCR in CAFs, as also found in cardiac fibroblasts (Snead and Insel, 2012). Multiple orphan and adhesion GPCRs are highly expressed in CAFs. GPR68, a proton-sensing GPCR, is much higher expressed in CAFs than in PSCs and has functional effects, including the production of IL6, which can promote the growth of PDAC cells (Wiley et al., 2018).

**FIGURE 8:**
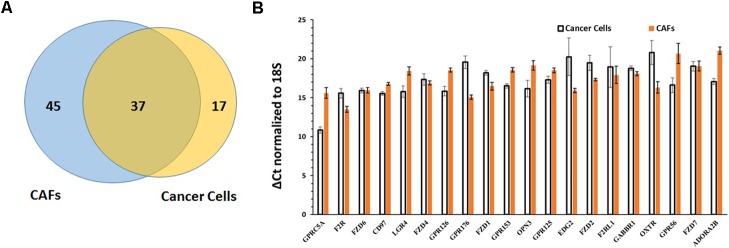
Comparison of GPCR expression in PDAC cells and CAFs. **(A)** PDAC cells and CAFs share in expression of 37 GPCRs. **(B)** The 20 GPCRs shared in expression by PDAC cells and CAFs and with the highest expression in PDAC cells and CAFs, determined by averaging GPCR expression in the two cell types. Mean and SEM are indicated for ΔCt values (normalized to 18S rRNA). Lower Δ Ct values indicate higher expression.

Comparison of the repertoires of 82 GPCRs commonly detected in CAFs and the 54 commonly detected GPCRs in PDAC cells revealed 37 GPCRs that were detected in both cell types (**Figure 8A**), among the highest expressed GPCRs in each cell type (**Figure 8B**), and often expressed at comparable magnitudes of expression. These commonly expressed GPCRs include certain orphan GPCRs (e.g., GPRC5A, CD97, GPR126) and GPCRs that are targets of approved drugs (e.g., OXTR, F2R/PAR1).

### Expression of Novel GPCRs (GPRC5A and GPR68) in PDAC Tumors and Cells

**FIGURE 9:**
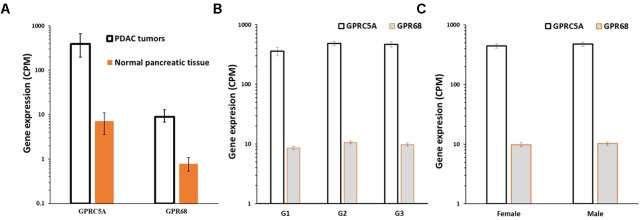
GPRC5A and GPR68 are highly expressed in PDAC tumors. **(A)** Median, upper and lower quartile expression values (indicated by the error bars) for GPRC5A and GPR68 in PDAC tumors (TCGA, *n* = 147) and in normal pancreatic tissue (GTEx, *n* = 165). In PDAC tumors, GPRC5A is increased ∼50-fold (FDR << 0.05) and GPR68 is increased ∼10-fold (FDR << 0.05). **(B)** Mean (and SEM) expression of GPRC5A and GPR68 in PDAC tumors (in TCGA) with tumor grades G1, G2, and G3. **(C)** Mean (and SEM) expression of GPRC5A and GPR68 in TCGA PDAC tumors from males and females. Expression differences for tumor grades and between males and females are not statistically significant.

Analysis of differential gene expression in PDAC tumors (TCGA) compared to normal pancreatic tissue (GTEx) revealed that GPRC5A and GPR68 are more highly expressed in the tumors than in normal tissue: GPRC5A is ∼50-fold increased in PDAC compared to normal pancreatic tissue, and GPR68 has ∼10-fold increased expression, each with a False Discovery Rate (FDR) << 0.05, indicating high statistical significance (**Figure 9A**). Both GPCRs lack evidence for dependence on factors such as patient’s sex and tumor grade (**Figures 9B,C**), suggesting that expression of these receptors increases early in tumor development and is highly prevalent in both male and female PDAC patients.

Data in the CCLE database support these findings regarding GPRC5A: it is the highest expressed GPCR in multiple PDAC cell lines [EBI gene expression atlas (Kapushesky et al., 2009) of results from cell lines in the CCLE (Barretina et al., 2012)]. GPRC5A is more highly expressed in every PDAC cell line with 31 of 33 PDAC cell lines having greater than twofold increased expression. GPRC5A is the highest expressed GPCR in 21/33 cell lines and among the three highest expressed GPCR in 30/33 cell lines.

**FIGURE 10:**
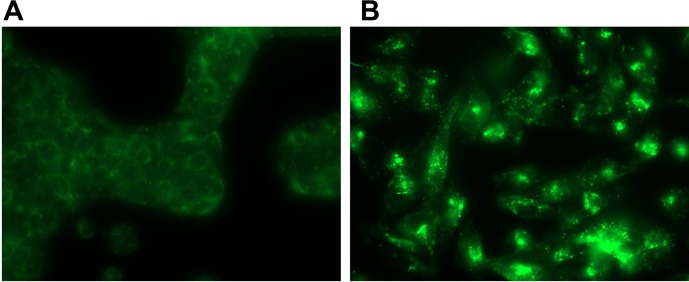
GPRC5A antibody staining in PDAC cells. **(A)** BXPC-3 **(B)** MIA PaCa-2. Using RNA-seq and qPCR, MIA PaCa-2 cells express >10-fold more GPRC5A mRNA than do BXPC-3 cells. Hence, MIA PaCa-2 cells stain more intensely for GPRC5A. Magnification = 10×. Images are representative of data for *n* = 3 separate slides for each cell line, respectively.

Immunocytochemistry staining of pancreatic cancer cells supports the mRNA expression data, indicating the presence of GPRC5A protein in these cells (**Figure 10**). Other data document the expression of GPRC5A using immunoblotting and immunohistochemistry in pancreatic cancer cells and tumors, respectively (Uhlén et al., 2015; Zhou et al., 2016, with relevant tumor staining data hosted at proteinatlas.org/ENSG00000013588-GPRC5A/pathology). In addition, other data show protein expression via immunocytochemistry and immunoblotting, which confirm the presence of GPR68 in pancreatic CAFs, and in PSCs in which GPR68 is overexpressed via transfection (Wiley et al., 2018).

## Discussion

The results shown here are, we believe, the first use of an unbiased (GPCRomic) approach to assess GPCR expression in the cells and tumors we studied. qPCR-based TaqMan GPCR arrays provide a means to identify and quantify GPCR mRNAs with a sensitivity and specificity superior to commercial tiling arrays that assess the entire transcriptome (Insel et al., 2015). Accordingly, gene expression data from the latter type of arrays (including data in public repositories) do not reveal an accurate or complete assessment of the GPCR repertoire of cancer cells. Improved detection by RNA-seq provides greater confidence regarding GPCR expression than tiling arrays but expression of GPCRs as a gene family has not previously been systematically culled out of RNA-seq data on cancer. The results here show that data from Taqman GPCR arrays and RNA-seq identify similar numbers of GPCRs in breast and colon cancers cells and in PDAC tumors and pancreatic CAFs. These assays hence provide complementary means of detection of GPCR expression in cancer cells and tumors.

A key conclusion from our studies is that each type of cancer cell/tumor expresses a common set of GPCRs. Some cell types and tumors express >150 different GPCRs, including ones expressed at relatively high levels. Many of the highly expressed receptors are orphan GPCRs. As we show for CLL and PDAC cells, multiple GPCRs are much more highly expressed by the malignant cells than by their normal precursors, B cells, and PDEC cells, respectively.

Certain cancer cell types may possess a “GPCR signature,” such that one or more GPCRs might serve as novel biomarkers and/or as therapeutic targets for such cancers. Therapeutic utility will require validation of the GPCRs preferentially expressed in cancer cells, including confirmation of expression of GPCR proteins, their signaling, and functional roles. Initial efforts indicate that at least certain of the GPCRs we have identified are functional in cancer cells [e.g., GPR161 in breast cancer (Feigin et al., 2014) and GPRC5A in pancreatic cancer (Zhou et al., 2016)] and in the microenvironment (e.g., GPR68; Wiley et al., 2018). These examples suggest that certain GPCRs are selectively overexpressed in specific cancer types, suggesting that different types of cancer may each possess a unique “GPCR-ome.” Certain GPCRs (e.g., CD97 and GPR56) are expressed in multiple cancer types, but also are widely expressed in normal tissue and cells (e.g., Uhlén et al., 2015, corresponding data for each GPCR hosted at proteinatlas.org). Besides the presence of these “promiscuously” expressed receptors, there does not appear to be a GPCR signature in common among cancer cells from different types of cancer.

In addition to cancer cells themselves, cells in the tumor microenvironment, as we show for BMNK cells and pancreatic CAFs, express a large number of GPCRs. Certain GPCRs, such as chemokine receptors, have been implicated in intercellular communication that can facilitate cancer cell proliferation, protection from apoptosis, and other features of the malignant phenotype (Hanahan and Weinberg, 2011; Vela et al., 2015; Guo et al., 2016). In addition, we recently documented a functional role for CAF-expressed GPR68 and its interaction with PDAC cells (Wiley et al., 2018).

Assessment of data in public databases has certain caveats, especially as related to reliability, reproducibility, and consistency of results from different labs, which may use different procedures and bioinformatics tools. We find that data derived from qPCR based arrays yield conclusions broadly supportive of those from analysis of public databases: Taqman array data for GPCR expression in PDAC tumors and cells that show GPRC5A is highly expressed are supported by TCGA data for PDAC tumors and RNAseq data from various cell lines (from the EBI portal). These complementary data suggest that while sources of technical variability may exist between datasets, the overall patterns of GPCR expression observed, and conclusions derived therefrom, are robust and reproducible.

As noted above, GPCRs with increased expression in cancer cells may be novel therapeutic targets. These include GPCRs for which approved drugs exist while other receptors identified include orphan, frizzled and/or adhesion GPCRs, which may provide unique therapeutic opportunities. GPCRs are commonly targeted by small molecules but other approaches may be possible, for example, techniques to blunt GPCR expression, GPCR-directed antibodies, antibody-drug conjugates, and bio-conjugates. (Junttila et al., 2015; Lelle et al., 2015; Gong et al., 2016; Gilabert-Oriol et al., 2017).

The approach described here – studies of patient-derived cells and cell lines to define a GPCR profile and the differential expression of GPCRs of cancer cells compared to that of normal cell precursors—should be applicable to other types of cancer. Perhaps therapies directed at such GPCRs can be part of therapeutic cocktails with multiple agents, especially since drug combinations may be able to optimize efficacy and minimize side-effects and drug resistance in cancer therapy (Atkins and Larkin, 2016; Alsaab et al., 2017; Corraliza-Gorjón et al., 2017; Gotwals et al., 2017; Lopez and Banerji, 2017).

## Author Contributions

PI, KS, HY, RC, SW, MF, and FM designed the experiments. AW, TK, TM, HY, LZ, SW, DL, and FM conducted experiments with GPCR arrays and analyzed data. AL and RF isolated and initially cultured pancreatic CAFs, PDAC, and PDEC cells and provided PDAC tumor samples. MF provided cDNA from breast cancer cell lines. KS performed immunocytochemistry, compiled and analyzed array data and mined data from databases. PI, KS, RC, and FM wrote the manuscript. All authors read and approved the manuscript.

## Conflict of Interest Statement

The authors declare that the research was conducted in the absence of any commercial or financial relationships that could be construed as a potential conflict of interest.

## Footnotes

1 xena.ucsc.edu

2 https://xenabrowser.net/datapages/?hub=https://toil.xenahubs.net:443

3 http://bioinformatics.mdanderson.org/tcgambatch/

4 https://nunofonseca.github.io/irap/

## Abbreviations

BMNK cell: Bone Marrow Natural Killer cell
CLL/B-CLL: Chronic Lymphocytic Leukemia/B-cell Chronic Lymphocytic Leukemia
Ct/ΔCt: Cycle threshold/Difference in Cycle threshold
EBI: European Bioinformatics Institute
FPKMs: Fragments Per Kilobase of transcript per Million mapped reads
GPCR: G protein-coupled receptor
GtoPdb: Guide to Pharmacology database
IUPHAR: International Union of Basic and Clinical Pharmacology
PDAC: Pancreatic Ductal Adenocarcinoma
PDEC: Pancreatic Ductal Epithelial Cell
PSCs: Pancreatic stellate cells
RNA-seq: High throughput RNA sequencing
TCGA: The Cancer Genome Atlas
TMM: trimmed mean of M values
